# UV Radiation and the Skin

**DOI:** 10.3390/ijms140612222

**Published:** 2013-06-07

**Authors:** John D’Orazio, Stuart Jarrett, Alexandra Amaro-Ortiz, Timothy Scott

**Affiliations:** 1 Graduate Center for Toxicology and the Departments of Pediatrics, Biomedical and Molecular Pharmacology and Physiology, Markey Cancer Center, University of Kentucky College of Medicine, 800 Rose Street, Lexington, KY 40536, USA; 2 Markey Cancer Center, University of Kentucky College of Medicine, 800 Rose Street, Lexington, KY 40536, USA; E-Mail: stuart.jarrett@uky.edu; 3 Graduate Center for Toxicology, University of Kentucky College of Medicine, 800 Rose Street, Lexington, KY 40536, USA; E-Mail: aalma2@uky.edu (A.A.-O.); tim.scott@uky.edu (T.S.)

**Keywords:** Ultraviolet radiation, skin, carcinogenesis, mutagenesis, pigmentation, cancer, melanin, melanocortin 1 receptor

## Abstract

UV radiation (UV) is classified as a “complete carcinogen” because it is both a mutagen and a non-specific damaging agent and has properties of both a tumor initiator and a tumor promoter. In environmental abundance, UV is the most important modifiable risk factor for skin cancer and many other environmentally-influenced skin disorders. However, UV also benefits human health by mediating natural synthesis of vitamin D and endorphins in the skin, therefore UV has complex and mixed effects on human health. Nonetheless, excessive exposure to UV carries profound health risks, including atrophy, pigmentary changes, wrinkling and malignancy. UV is epidemiologically and molecularly linked to the three most common types of skin cancer, basal cell carcinoma, squamous cell carcinoma and malignant melanoma, which together affect more than a million Americans annually. Genetic factors also influence risk of UV-mediated skin disease. Polymorphisms of the melanocortin 1 receptor (*MC1R*) gene, in particular, correlate with fairness of skin, UV sensitivity, and enhanced cancer risk. We are interested in developing UV-protective approaches based on a detailed understanding of molecular events that occur after UV exposure, focusing particularly on epidermal melanization and the role of the MC1R in genome maintenance.

## 1. The Skin

**Figure 1:**
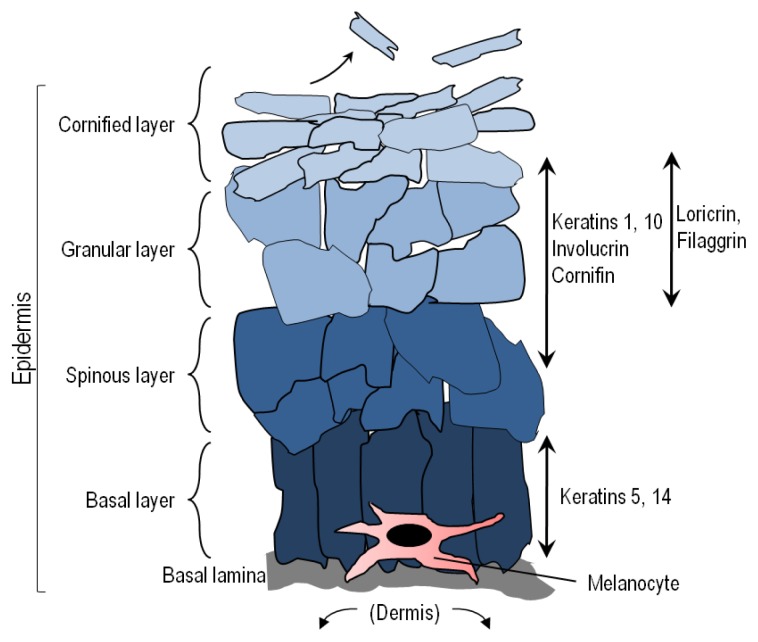
Epidermal structure and keratinocyte differentiation. The epidermis is a self-renewing tissue composed mainly of keratinocytes in various stages of terminal differentiation. Keratinocytes are produced in the stratum basale (basal layer), and move outward through the epidermis, undergoing a programmed series of differentiation involving enucleation and accumulation of cytokeratins and tight junctions with each other. Keratinocytes also receive melanin from melanocytes in the form of pre-packaged organelles termed melanosomes. The basic layers from the basement membrane outward are the stratum basale, stratum spinosum, stratum granulosum, and the stratum corneum, each identified by the morphology and differentiation state of the keratinocyte as indicated by expression of cytokeratins and other proteins.

Comprising roughly 16% of body mass, the skin is the largest organ of the body. Skin is organized into two primary layers, epidermis and dermis, which together are made up of epithelial, mesenchymal, glandular and neurovascular components. The epidermis, of ectodermal origin, is the outermost layer and serves as the body’s point of contact with the environment. As such, epidermal biological and physical characteristics play an enormous role in resistance to environmental stressors such as infectious pathogens, chemical agents and UV [1–6]. Keratinocytes are the most abundant cells in the epidermis and are characterized by their expression of cytokeratins and formation of desmosomes and tight junctions with each other to form an effective physicochemical barrier. The dermis, derived from mesoderm, underlies the epidermis and harbors cutaneous structures including hair follicles, nerves, sebaceous glands and sweat glands. The dermis also contains abundant immune cells and fibroblasts, which actively participate in many physiologic responses in the skin. The epidermis, demarcated from the dermis by a basement membrane, is organized into functional layers defined largely by keratinocyte characteristics such as size, shape, nucleation and keratin expression [7] (Figure 1). Nascent epidermal keratinocytes formed as a result of cell division by keratinocyte stem cells in the stratum basale undergo a programmed differentiation as they migrate outward toward the surface of the skin to eventually form corneocytes, which are tightly-linked dead but intact cells that form the principle barrier of the outermost epidermal layer [8,9].

Besides the creation of a highly effective physical barrier, keratinocytes also accumulate melanin pigments as they mature, and epidermal melanin functions to potently block UV penetration into the skin. Although melanin may be found in abundance in epidermal keratinocytes, it is not manufactured in these cells. Rather, melanin synthesis is restricted to melanocytes, which are derived from neural crest and are the second most abundant cell in the epidermis [10,11]. In fact, melanocytes can be found both in the dermis and epidermis. Epidermal melanocytes are generally positioned in the basal layer above the basement membrane. Melanocytes are also found in hair follicles to impart pigment to nascent hair [12]. Dermal melanocytes can be found in nevi (moles). Because melanocytes are the only source of pigment in the skin, inherited pigmentary defects such as albinism tend to be caused by melanocytic genetic defects [10,13]. Through dendritic extensions, melanocytes may be in intimate contact with as many as fifty neighboring keratinocytes in what is known as an “epidermal melanin unit” [11,14]. There are many contact-dependent and paracrine interactions that occur between keratinocytes and melanocytes in the epidermal melanin unit. Pigment made by melanocytes is transferred to adjacent keratinocytes in cellular organelles termed melanosomes by way of melanocytic dendrites [15–17]. In fact, most of the melanin in the skin is found in keratinocytes where it accumulates to function as a “natural sunscreen” to protect the skin against incoming UV photons. Besides blocking UV penetration into the skin, melanin may have many other important physiologic effects including regulatory influences over epidermal homeostasis, free radical scavenging to protect against oxidative injury, and possibly even antimicrobial activity [10,18–24].

## 2. Melanin

**Figure 2:**
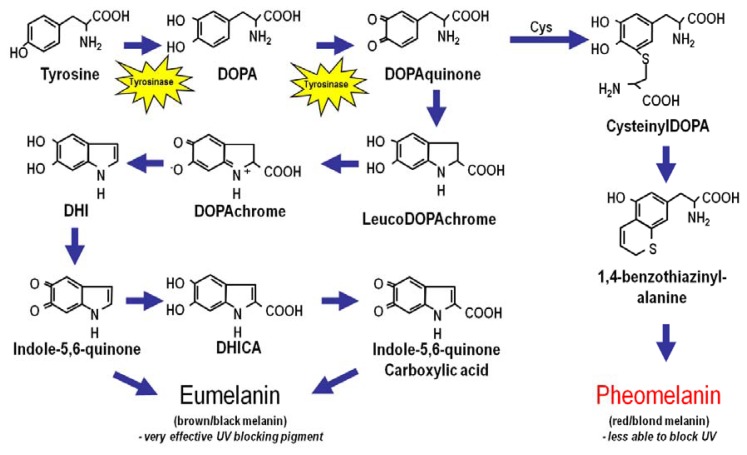
Melanin Biosynthesis. Melanin, a large bioaggregate composed of pigmented chemical species, is found in two major forms: the brown/black highly UV-protective “eumelanin” pigment and the red/blonde UV-permeable “pheomelanin”. Both eumelanin and pheomelanin are derived from the amino acid tyrosine. Tyrosinase is the enzyme that catalyzes the rate-limiting synthetic reaction for both melanin species and when defective causes albinism. Incorporation of cysteine into pheomelanin results in the retention of sulfur into the pigment, which yields a light color to the final melanin product and may contribute to oxidative injury in the skin. The melanocyte stimulating hormone (MSH)–melanocortin 1 receptor (MC1R) signaling axis is a major determinant of the type and amount of melanin produced by melanocytes in the skin.

The amount and type of epidermal melanin is the main factor that determines skin complexion and UV sensitivity. Melanin is a large bio-aggregate composed of subunits of different pigment species formed by oxidation and cyclization of the amino acid tyrosine [10,25,26] (Figure 2). Intriguingly, the intermediates of melanogenesis may have important regulatory roles in the skin [27–29]. Melanin exists in two main chemical forms: (1) eumelanin, a dark pigment expressed abundantly in the skin of heavily pigmented individuals, and (2) pheomelanin, a light-colored sulfated pigment resulting from incorporation of cysteines into melanin precursors [30]. Eumelanin is much more efficient at blocking UV photons than pheomelanin, thus the more eumelanin in the skin, the less UV-permeable is the epidermis [31]. Fair-skinned people who are almost always UV-sensitive and have high risk of skin cancer have little epidermal eumelanin and therefore “realize” much more UV than darker-skinned individuals. Therefore, the fairer the skin, the more damaging UV exposure will be. In fact, pheomelanin levels are similar between dark-skinned and light-skinned individuals, and it is the amount of epidermal eumelanin that determines skin complexion, UV sensitivity and cancer risk. Data suggest that pheomelanin may promote oxidative DNA injury and melanomagenesis by generating free radicals in melanocytes even in the absence of UV [32–37].

## 3. Skin Pigmentation

Skin complexion is among the most important determinants of UV sensitivity and skin cancer risk. The “Fitzpatrick Scale” is a semi-quantitative scale made up of six phototypes that describe skin color by basal complexion, melanin level, inflammatory response to UV and cancer risk [13] (Table 1). Minimal erythematous dose (MED) is a quantitative method to report the amount of UV (particularly UVB) needed to induce sunburn in the skin 24–48 h after exposure by determining erythema (redness) and edema (swelling) as endpoints. The fairer the skin, the easier it is for UV to cause inflammation (sunburn). MED, therefore is highest in dark-skinned persons since more UV radiation is needed to “burn” eumelanin-rich skin [38–40]. In contrast, fair-skinned people whose skin expresses predominantly pheomelanin have low MEDs. Low Fitzpatrick phototype correlates with both MED and with melanoma and other skin cancer risk [41].

## 4. Ultraviolet Radiation (UV)

**Figure 3:**
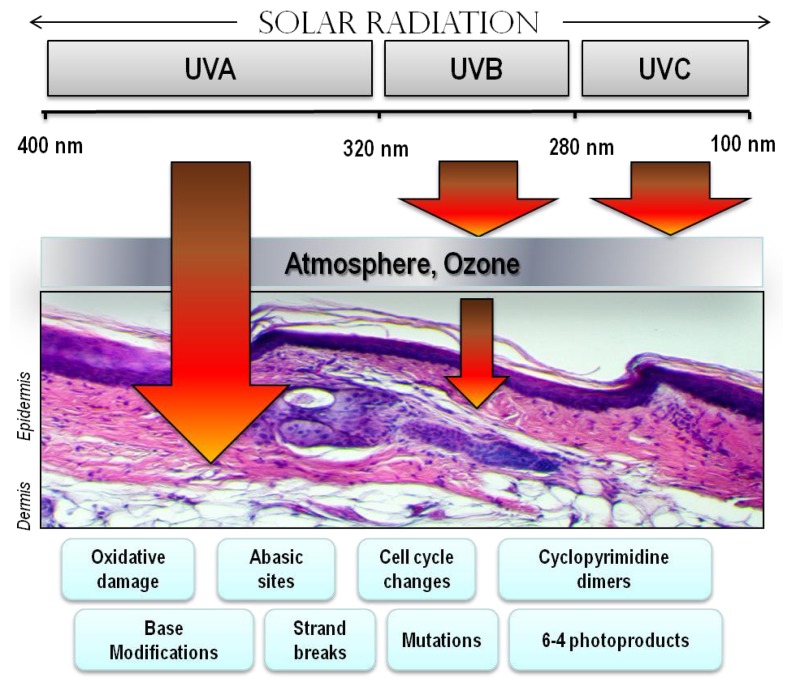
Electromagnetic spectrum of visible and UV radiation and biologic effects on the skin. Solar UV radiation can be subdivided into UVA, UVB and UVC components, however because of atmospheric ozone that absorbs UVC, ambient sunlight is predominantly UVA (90%–95%) and UVB (5%–10%). UV penetrates the skin in a wavelengthdependent manner. Longer wavelength UVA penetrates deeply into the dermis reaching well into the dermis. In contrast, UVB is almost completely absorbed by the epidermis, with comparatively little reaching the dermis. UVA is efficient at generating reactive oxygen species that can damage DNA via indirect photosensitizing reactions. UVB is directly absorbed by DNA which causes molecular rearrangements forming the specific photoproducts such as cyclobutane dimers and 6–4 photoproducts. Mutations and cancer can result from many of these modifications to DNA.

Abundant in the environment, UV contributes to a variety of skin maladies including inflammation, degenerative aging and cancer [1]. Historically, humans have been exposed to UV radiation mainly through occupational exposure to sunlight. Recreational UV exposure, however, has increased dramatically in recent years because of outdoor leisure activities and to purposely tan for cosmetic purposes [42,43]. Being a component of the electromagnetic spectrum, UV photons fall between the wavelengths of visible light and gamma radiation. UV energy can be subdivided into UV-A, -B and -C components based on electro physical properties, with UV-C photons having the shortest wavelengths (100–280 nm) and highest energy, UV-A having the longest (315–400 nm) but least energetic photons and UV-B falling in between (Figure 3). Each component of UV can exert a variety of effects on cells, tissues and molecules.

Ambient UV exposure varies geographically according to intensity of sunlight in a particular location on Earth. Since UV radiation can be reflected, scattered and dampened by atmospheric particles, ambient UV dose varies according to the amount of atmosphere it must pass through, making UV doses higher nearest the Equator (where sunlight strikes the Earth most directly), at higher altitudes and in conditions of minimal cloud or particulate cover. Personal UV dosing depends not only on strength of solar radiation, but also on time spent outdoors occupationally or recreationally and the usage of UV-protective clothing, shade and sun blocks. Since equatorial locations tend to be warm and conducive to recreational or occupational outdoor activities, people living such locales typically wear less clothing and have more contact with ambient sunlight and usually receive much higher ambient UV doses than persons inhabiting temperate climates. Not surprisingly, skin cancer risk generally mirrors this geographic pattern, particularly among fair-skinned sun-sensitive persons [44–46].

## 5. Indoor Tanning

The number and use of indoor tanning salons has skyrocketed over the last several years. In America alone, only 1% of the population had ever used a tanning bed in the late 1980s. Now it is estimated that over 25% of Americans have engaged in purposeful exposure to artificial UV radiation [47]. Indoor tanning is an important industry with nearly 30 million clients, 100,000 employees and billions of dollars of annual business. Indoor tanning machines are poorly regulated and vary widely with respect to UV composition and strength. UV output from tanning beds can be up to ten times more powerful than sunlight [48,49], making the tanning bed an authentic carcinogenic instrument. Tanning can be addictive, leading to frequent and significant UV exposure over time [50–52], and since tanning often appeals to adolescents and young adults, tanning patrons’ UV history can be significant for many years [53].

**Figure 4:**
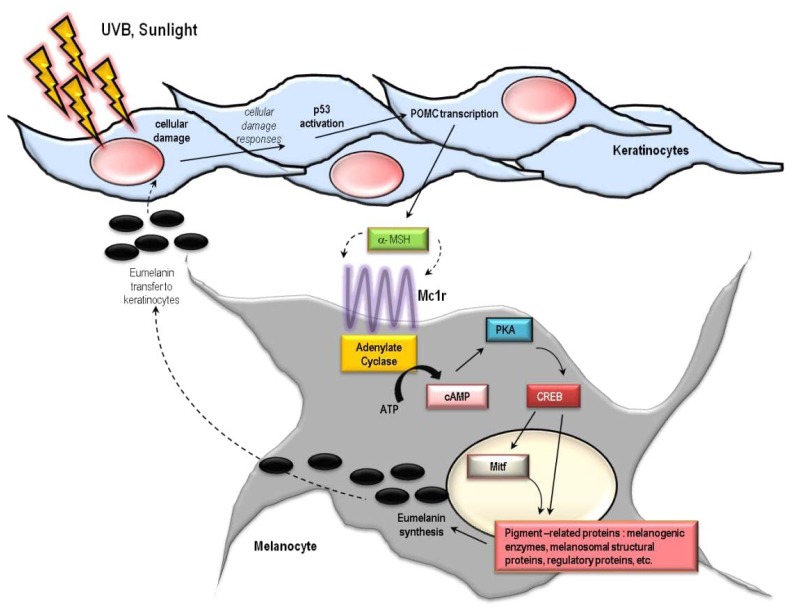
Mechanisms of the physiologic tanning response. Hormonal interactions between epidermal keratinocytes and melanocytes mediate much of the cutaneous melanization response. DNA and cellular damage in keratinocytes up-regulates transcription of the pro-opiomelanocortin (POMC) gene which encodes production and secretion of melanocyte stimulating hormone (α-MSH). α-MSH binding to melanocortin 1 receptor (MC1R) on melanocytes in the basal epidermis generates the second messenger cAMP via interactions between MC1R and adenylyl cyclase, and leads to activation of protein kinase A and the cAMP responsive binding element (CREB) and microphthalmia (Mitf) transcription factors. CREB and Mitf directly enhance melanin production by raising levels of tyrosinase and other melanin biosynthetic enzymes. Thus, MSH-MC1R signaling leads to enhanced pigment synthesis by melanocytes and accumulation of melanin by epidermal keratinocytes. By this mechanism, the skin is better protected against UV insults. Of note, UV-induced pigmentation may also occur through other signaling pathways as well as direct effects of UV on melanocytes, and there is some disagreement in the field over the role of epidermal MSH in the adaptive pigmentary response.

Indoor tanning clearly increases incidence of skin cancers [54,55]. With respect to melanoma, the deadliest of skin malignancies, lifetime risk increases by 75% if people engage in artificial tanning before the age of 35 years [56–58]. Cancer risk increases with years of use, number of sessions, and total number of UV h exposed [54,56,59,60]. Since the molecular pathways in the skin that activate UV-induced tanning result from cellular and DNA damage which underlie skin damage and carcinogenesis (Figure 4), it appears as though there is no “safe” use of tanning salons [57]. The tanning industry has engaged a powerful political lobby to further its commercial interests by downplaying the adverse health risks of UV. Instead, the industry publicizes the health benefits of UV to its clients, emphasizing vitamin D production which is naturally made in the skin by the chemical conversion of 7-dehydrocholesterol into vitamin D_3_ (cholecalciferol) after UVB exposure [61–69]. In fact, UV doses that induce tanning far exceed what is required for adequate vitamin D production and the widespread availability of vitamin D in supplements and fortified foods minimizes the need for UV exposure to avoid symptoms of rickets and vitamin D deficiency [70–74]. Multiple studies report overwhelming evidence that the risks of indoor tanning far outweigh potential health benefits, most significantly for malignancy. Decreasing UV radiation exposure, both naturally from sunlight and artificially from tanning bed use, may be the single best way to reduce incidence of melanoma and other skin cancers [75].

## 6. Cutaneous Responses to UV

UV has many effects on skin physiology, with some consequences occurring acutely and others in a delayed manner. One of the most obvious acute effects of UV on the skin is the induction of inflammation. UVB induces a cascade of cytokines, vasoactive and neuroactive mediators in the skin that together result in an inflammatory response and causes “sunburn” [3,4,6,76–79]. If the dose of UV exceeds a threshold damage response, keratinocytes activate apoptotic pathways and die. Such apoptotic keratinocytes can be identified by their pyknotic nuclei and are known as “sunburn cells” [80]. UV also leads to an increase in epidermal thickness, termed hyperkeratosis. By causing cell injury, UV induces damage response pathways in keratinocytes. Damage signals such as p53 activation profoundly alter keratinocyte physiology, mediating cell cycle arrest, activating DNA repair and inducing apoptosis if the damage is sufficiently great. Several h after UV exposure, however, and damage response signals abate, epidermal keratinocytes proliferate robustly [81], mediated by a variety of epidermal growth factors. Increased keratinocyte cell division after UV exposure leads to accumulation of epidermal keratinocytes which increases epidermal thickness. Epidermal hyperplasia protects the skin better against UV penetration [82].

Coupled with epidermal hyperkeratosis is adaptive melanization of the skin, also known as tanning [4,10,83–86]. UV up-regulates production and epidermal accumulation of melanin pigment in the skin [87–91]. This important physiologic response protects the skin against subsequent UV damage, and defects in this pathway are linked with cancer susceptibility. UV-mediated skin darkening is actually biphasic, with initial skin darkening occurring from redistribution and/or molecular changes to existing epidermal melanin pigments. Delayed increases in skin darkening, mediated by actual up-regulation in melanin synthesis and transfer to keratinocytes, begin several h to days after UV exposure [92,93]. Adaptive melanization is likely a complex physiologic response [4,10,83,85] involving multiple skin cell types interacting in a variety of ways (Figure 4) [86,94–102]. UV has many other effects on the skin, including induction of an immune-tolerant or immunosuppressive state [103–110] and production of vitamin D by direct conversion of 7-dehydrocholesterol into vitamin D_3_ (cholecalciferol) [61–69]. Ambient sunlight, for the most part, is a mixture of UVA and UVB, yet each UV component may exert different and distinct effects on the skin [111,112]. UVB, for example, is a potent stimulator of inflammation and the formation of DNA photolesions (such as mutagenic thymine dimers) [112,113], whereas UVA is much less active in these measures but instead is a potent driver of oxidative free radical damage to DNA and other macromolecules [114–116]. Thus, each may contribute to carcinogenesis through different mechanisms [117–119]. The influence of UVA and UVB on skin physiology is an active area of investigation.

## 7. Oxidative Injury

**Figure 5:**
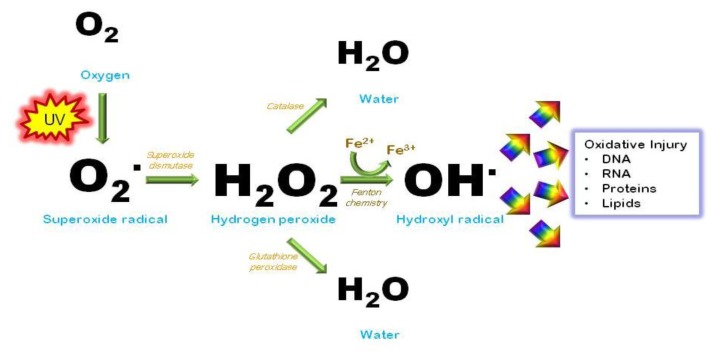
UV generates oxidative free radicals. UV photons interact with atomic oxygen to promote formation of free radical derivatives such as superoxide, hydrogen peroxide and the highly reactive hydroxyl radical. Free radicals avidly attack macromolecules such as protein, lipid, RNA and DNA, altering their structure and interfering with their function. Detoxifying and protective enzymes such as superoxide dismutase, catalase and glutathione peroxidase detoxify and reduce levels of oxidative species in the cell.

Besides promoting formation of photodimers in the genome, UV causes mutations by generating reactive oxygen species (ROS) such as superoxide anion, hydrogen peroxide and the hydroxyl radical [21] (Figure 5). Nucleotides are highly susceptible to free radical injury. Oxidation of nucleotide bases promotes mispairing outside of normal Watson-Crick parameters, causing mutagenesis [120]. The transversion guanine→thymine, for example, is a well-characterized mutation caused by ROS by oxidizing guanine at the 8th position to produce 8-hydroxy-2′-deoxyguanine (8-OHdG) [121,122]. 8-OHdG tends to pair with an adenine instead of cytosine and therefore this oxidative change mutates a G/C pair into an A/T pair. Such mutations can be found in tumors isolated from the skin, suggesting that oxidative injury can be carcinogenic [123]. Cellular maintenance pathways exist to inactivate oxidative species as well as to repair the DNA damage they cause. The base excision repair pathway (BER) is the main molecular means by which cells reverse free radical damage in DNA to avoid oxidative mutagenesis. This pathway is initiated by damage-specific glycosylases that scan DNA for specific alterations including deaminated, alkylated or oxidized bases. After altered or inappropriate bases are recognized by a lesion-specific glycosylase, the enzyme cleaves the nucleotide base from the sugar and phosphodiesterase backbone by lysis of the *N*-glycosylic bond between the base and the deoxyribose. This step forms an abasic or apurinic/apyrimidinic (AP) site in the DNA, which is then processed and repaired using the complementary strand as a template to ensure fidelity.

Cells also have a complex and robust network of anti-oxidant molecules that detoxify reactive species to prevent free radical changes to DNA and other macromolecules. Glutathione (GSH) is an oligopeptide made up of three amino acids- cysteine, glycine and glutamine and is among the most important cellular antioxidant molecules. By donating electrons to otherwise reactive molecules, GSH functions as a reducing agent to neutralize reactivity of free radicals. In the process, glutathione itself becomes oxidized but can be reduced to its basal state by glutathione reductase using NADPH as an electron donor and be recycled. In any cell, therefore, glutathione can be found in both its reduced and oxidized forms and abnormalities in the ratio of reduced to oxidized glutathione can indicate oxidative stress. Catalase is another major antioxidant enzyme that detoxifies hydrogen peroxide [124–126], whereas superoxide dismutases (SOD’s) inactivate superoxide anions [127]. Regulation of these antioxidant enzymes is a major area of investigation [128,129] since it is critical in determining cutaneous responses to UV radiation.

## 8. Nucleotide Excision Repair and Xeroderma Pigmentosum

Besides free radical formation, UV directly affects nucleotide base pairing in DNA [130,131]. Pyrimidine bases are particularly vulnerable to chemical alteration by absorption of UV energy. Shorter-wavelength UV photons, particularly UV-B and UV-C, cleave internal 5–6 double bonds of pyrimidines. When this occurs between adjacent pyrimidines, abnormal covalent bonds may form and alter the three-dimensional structure of the double helix. Two major photolesions- cyclobutane pyrimidine dimers or (6,4)- photoproducts- predictably form in this way after UV exposure, and both are highly mutagenic [132]. It is estimated that one day’s worth of sun exposure results in up to 10^5^ UV-induced photolesions in every skin cell [133]. UV-induced photolesions impair transcription, block DNA replication and base pair abnormally. They cause characteristic transition mutations known as “UV signature mutations”, for example, TT→CC. The abundance of UV signature mutations in cancer-regulatory genes among many primary skin cancer isolates strongly supports UV as a cancer-causing agent [134–137].

**Figure 6:**
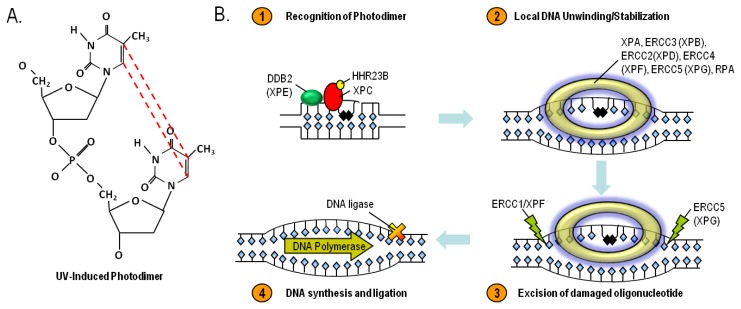
UV-induced cyclobutane dimers- structure (**A**) and repair by the Nucleotide Excision DNA Repair (NER) pathway (**B**). The NER pathway is mediated by at least eight enzymes that work together to identify bulky DNA lesions that distort the structure of the double helix, excise the damaged portion and replace the excised region by DNA synthesis directed by the complementary strand. Homozygous deficiency in any one of the NER enzymes leads to the clinical condition known as Xeroderma Pigmentosum (XP). Although not shown, NER can also be initiated in actively transcribed regions of the genome by involvement of the Cockayne syndrome proteins A and B.

Nucleotide excision repair (NER) is an evolutionarily-conserved mechanism for repairing UV-induced photoproducts and other bulky DNA lesions [138]. The importance of NER in cancer resistance is best illustrated by considering the natural history of patients with Xeroderma Pigmentosum (XP), a rare UV hypersensitivity syndrome caused by homozygous defects in any one of at least eight required effector proteins of a common pathway that executes NER: *XPA*, *ERCC1*, *ERCC3 (XP-B)*, *XPC*, *ERCC2 (XP-D)*, *DDB2 (XP-E)*, *ERCC4 (XP-F)*, *ERCC5 (XP-G)* and *POLH*. XP patients demonstrate profound UV sensitivity and develop characteristic skin changes including pigmentary abnormalities, capillary telangiectasias and atrophy on UV-exposed anatomic sites at very early ages. Premalignant lesions and skin cancers develop in high frequency and much sooner than in unaffected persons. Basal cell carcinomas, squamous cell carcinomas and melanomas often develop before the second decade of life, decades before the general population [139]. Moreover, XP-associated skin cancers frequently demonstrate “UV signature mutations”, clearly indicating the importance of NER in the cancer resistance [140]. The NER pathway represents an orchestrated interaction of enzymes that function together to repair lesions that alter the three-dimensional structure of DNA. After recognition of damage and recruitment of a multiprotein repair complex to the damaged site, the damage strand is nicked several nucleotides away on either side of the damaged bases. The damaged region is excised and the resulting gap is filled in by a DNA polymerase using the non-damaged strand as a template [141–143] (Figure 6). Though only a handful of core factors are necessary and sufficient for the repair of UV-induced DNA lesions, there are numerous accessory factors that regulate this genome maintenance pathway. While the importance of NER in UV and skin cancer resistance is most clearly demonstrated by the natural history of patients with XP, attention is being paid to the role of NER polymorphisms on UV sensitivity and skin cancer incidence in sporadic populations.

## 9. Skin Cancer

**Figure 7:**
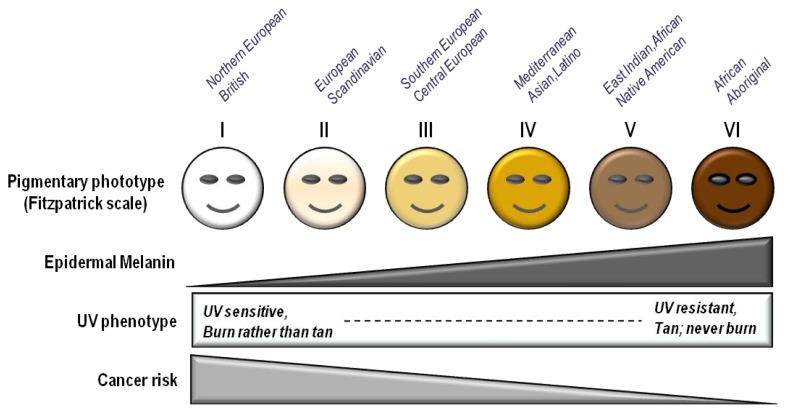
Influence of pigmentation on skin cancer risk. Fair-skinned individuals with low levels of melanin in the epidermis display a UV sensitive phenotype, tending to burn rather than tan, after UV exposure. Recent data suggest that mutations that contribute to fair complexion and tanning impairment, specifically signaling defects in the melanocortin 1 receptor (MC1R), may also be associated with less efficient DNA repair in melanocytes. MC1R-defective individuals not only suffer higher realized doses of UV radiation because their skin is less able to block UV photons, but they may also accumulate more mutations from UV exposure because of defective DNA repair.

Skin cancers are by far the most common malignancies of humans, with well over a million cases diagnosed each year [144]. Roughly 1 in 5 Americans will develop skin cancer in their lifetime [145]. They account for nearly 15,000 deaths and more than three billion dollars each year in medical costs in the United States alone [146,147]. Like many other cancers contributed to by environmental etiologies (in this case UV), skin cancer incidence increases markedly with age presumably reflecting the long latency between carcinogen exposure and cancer formation. Skin cancers are commonly grouped into two main categories, melanoma and non-melanoma skin cancers (NMSC), based on cell of origin and clinical behavior. Risk of skin cancer is heavily influenced by UV exposure and by skin pigmentation [148] (Figure 7).

Malignant melanoma of the skin is the deadliest form of skin cancer. Thought to arise from epidermal melanocytes, melanoma is a treatment-refractory and metastasis-prone malignancy whose incidence has increased steadily and significantly over the last several decades [149]. Whereas only one in 1500 Americans was ever diagnosed with melanoma in the 1930s, now roughly one in sixty will be affected by the disease [150]. Melanoma accounts for about three quarters of all deaths from skin cancers, numbering nearly ten thousand per year in the U.S., despite accounting for far fewer than ten percent of all skin malignancies. Melanoma burden is predictably largest in places with large numbers of fair-skinned individuals living in warm, sunny climates [151]. Most melanomas arise out of pre-existing moles, therefore having many nevi is another important risk factor for the disease. If caught early, many melanomas can be managed by surgical excision alone. However, melanomas are quick to invade and metastasize and long-term survival is poor for advanced disease. Even with recent progress made in targeted therapy [152–156] and immunotherapy [157,158], melanoma is notoriously difficult to treat once it has spread beyond its original site. It is not clear why melanoma incidence has increased so dramatically over the past several decades, but it is likely multifactorial, with contributions from increased UV exposure, environmental and inherited cancer risk factors and better surveillance and earlier detection [151,159–172].

Non-melanomatous skin cancers greatly outnumber melanomas in incidence, but fortunately most are much easier to treat and have much better long-term prognosis. The two major forms, basal cell carcinomas and squamous cell carcinomas, are both derived from epidermal keratinocytes. They are less deadly than melanoma mainly due to their tendency to remain confined to their primary site of disease, which makes their management much more straightforward. The overwhelming majority of keratinocyte malignancies develop in the areas of skin most exposed to UV, such as on the face and arms. Most are effectively treated by local control measures alone such as resection, MOHS microsurgery or cryosurgery.

There are strong epidemiologic and molecular data linking all forms of skin cancer to UV exposure [173], and it is estimated that UV is causative for nearly 65% of melanoma and 90% of non-melanoma skin cancers [174,175]. UV-signature mutations in key cancer-relevant genes such as the p53 tumor suppressor in squamous cell carcinoma for example are well-characterized, and exome analysis of a panel of melanomas revealed strong genetic evidence for a direct mutagenic role of UV radiation in the pathogenesis of melanoma [137,176–183]. Since UV-induced DNA mutations represent a major causative factor for melanoma and other skin cancers, it follows that resistance to UV-mediated mutagenesis is a critical determinant of skin cancer risk [184].

## 10. The Melanocortin 1 Receptor (MC1R)

The melanocortin 1 receptor (MC1R) is a critical genetic locus involved in pigmentation, the adaptive tanning response and skin cancer susceptibility [185–192]. The MC1R is found on the surface of melanocytes where it binds to α-melanocyte stimulating hormone (MSH) and transmits differentiation signals into the cell through activation of adenylyl cyclase and generation of cAMP [193–195]. cAMP signaling leads to activation of the protein kinase A (PKA) cascade which, in turn, leads to increased levels and/or activity of many melanogenic enzymes to enhance production and export of melanin by melanocytes [90,196,197] (Figure 4). MC1R signaling also decreases UV-mediated mutagenesis by enhancing genome maintenance pathways in melanocytes [125,126,192,198]. Loss-of-signaling MC1R polymorphisms are commonly found among fair-skinned, sun-sensitive and skin cancer-prone populations (e.g., Northern Europeans). The most prevalent MC1R mutations (D84E, R151C, R160W and D294H) are commonly referred to as “RHC” (red hair color) alleles because of their association with red hair color, freckling and tendency to burn after UV exposure [199,200]. Loss of signaling MC1R alleles such as the RHC variants are associated with up to a four-fold increased lifetime risk of melanoma and other skin cancers [201–203]. Overall, there is much evidence placing MC1R as a critical determinant of skin cancer risk, and regulation of eumelanin by POMC derived peptides depends on genetic context [204].

MC1R signaling protects the skin from UV damage by at least two major mechanisms. First, by inducing pigment synthesis in melanocytes, MC1R enhance production and accumulation of eumelanin in the epidermis. Epidermal melanization blocks penetration of UV into the skin, reducing realized doses of UV and decreasing mutagenesis and cancer risk. MC1R signaling also directly influences UV resistance of melanocytes by enhancing nucleotide excision DNA repair and oxidative resistance. Since MC1R signaling is potentially targetable by agents that influence cAMP levels [82,84,205], pharmacologic manipulation of cutaneous cAMP may be a useful approach to reduce UV sensitivity and cancer risk. Theoretically, raising cAMP levels in the skin can be accomplished either by stimulating its production (e.g., adenylyl cyclase activation) or by impeding its degradation (e.g., phosphodiesterase inhibition). Both of these approaches have been quite successful in enhancing epidermal melanin levels in animal models [84,206] and each would be expected to be effective even in individuals harboring loss-of-signaling functional mutations in MC1R. Alternatively, α-MSH or agonistic MC1R peptide ligands would offer more specificity (working only on melanocytes) but might be less effective in individuals with inherited MC1R signaling defects [192,193,207].

## 11. Conclusions

One of the greatest risk factors for the development of cutaneous melanoma is having a fair skin complexion, which is characterized by low levels of a UV-blocking dark pigment called eumelanin in the epidermis. Individuals with light skin pigmentation suffer comparatively more skin damage from UV because it is relatively easy for UV rays to penetrate the epidermis to damage both keratinocytes and melanocytes in the deeper layers of the epidermis. Fair-skinned individuals are exposed to higher “realized” doses of UV radiation in the skin and UV-induced mutations, which directly contribute to melanoma and other forms of skin cancer, accumulate over time. Much UV-induced pathology, including skin cancer, can be avoided by minimizing UV exposure (Table 2).

We and others are increasingly interested in heritable factors that determine melanoma risk to be able to intervene in the carcinogenic process. One of the most important alleles that influences skin cancer risk is the melanocortin 1 receptor (MC1R), whose function is central to the adaptive pigmentation (tanning) response in the skin. Besides mediating the tanning response, MC1R exerts a powerful influence on the ability of melanocytes to repair UV-induced DNA damage by the nucleotide excision DNA repair pathway. New insights into the ways in which MC1R and other genes function to protect the skin against the harmful consequences of UV may allow the rational development of pharmacologic strategies to reduce UV sensitivity and cancer risk.

## Figures and Tables

**Table 1:** Skin pigmentation, the Fitzpatrick scale and UV risk.

Fitzpatrick phototype	Phenotype	Epidermal eumelanin	Cutaneous response to UV	MED (mJ/cm^2^) *	Cancer risk
I	Unexposed skin is bright white Blue/green eyes typical Freckling frequent Northern European/British	+/−	Always burns Peels Never tans	15–30	++++
II	Unexposed skin is white Blue, hazel or brown eyes Red, blonde or brown hair European/Scandinavian	+	Burns easily Peels Tans minimally	25–40	+++/++++
III	Unexposed skin is fair Brown eyes Dark hair Southern or Central European	++	Burns moderately Average tanning ability	30–50	+++
IV	Unexposed skin is light brown Dark eyes Dark hair Mediterranean, Asian or Latino	+++	Burns minimally Tans easily	40–60	++
V	Unexposed skin is brown Dark eyes Dark hair East Indian, Native American, Latino or African	++++	Rarely burns Tans easily and substantially	60–90	+
VI	Unexposed skin is black Dark eyes Dark hair African or Aboriginal ancestry	+++++	Almost never burns Tans readily and profusely	90–150	+/−

Minimal erythematous dose (MED) is defined as the least amount of UVB radiation that causes reddening and inflammation of the skin 24–48 h after exposure (*i.e.*, the lowest UV dose that causes sunburn). The more UV sensitive an individual is, the lower the MED of his/her skin.

**Table 2:** UV Safety Tips.

**Sun exposure**	Minimize time outdoors during “peak” UV h (10 am to 4 pm). Seek shade as much as possible. Be aware that sunlight bounces off reflective surfaces and can reach you even under an umbrella or tree. Avoid getting a sunburn. More than 5 sunburns doubles risk of skin cancer. Use sunscreens with a sun protection factor (SPF) >15. Make sure to apply repeatedly (especially with sweating or swimming) and as directed. Use sunblocks that offer protection from both UV-A and UV-B rays, and be sure to cover often-missed spots- lips, ears, around eyes, neck, scalp, hands and feet. Wear protective clothing such as rash guards and tightly woven fabrics. Wear a hat. Wide-brimmed hats protect head, face, ears and neck. If a baseball cap is worn, make sure to use sunscreen on ears and neck. Wear UV-protective sunglasses Strength of solar UV increases at high altitude and with less cloud cover. Monitor the UV Index (http://www.epa.gov/sunwise/uvindex.html) and plan accordingly. Get Vitamin D safely by relying on diet and supplements rather than UV exposure.
**Artificial Tanning**	Do not frequent tanning beds. They can be more dangerous than sunlight. Frequent use of artificial tanning products clearly increases risk of each of the major kinds of skin cancer, including melanoma. Sunless self-tanning products seem safe but typically offer little sun-blocking UV protection on their own.
**Awareness**	Examine your skin frequently, at least once a month, head to toe. Use a full-length mirror and a hand mirror to check your back, or involve a partner. Have a professional skin examination annually. Seek professional medical attention for: Sores that do not heal Changes in moles (growth, irregularity, asymmetry, color changes, elevation, pain, itching) Skin cancers are much more easily treated when caught early.
